# Use of Video-Enabled Tablet for Virtual Care Among Older Veterans

**DOI:** 10.1093/geroni/igaa057.637

**Published:** 2020-12-16

**Authors:** Maria Yefimova, Jiaqi Hu, Cindie Slightam, Liberty Greene, Camila Chaudhary, Leonie Hayworth, Donna Zulman

**Affiliations:** 1 VA Palo Alto Healthcare System, Redwood City, California, United States; 2 VA Palo Alto Healthcare System, Palo Alto, California, United States; 3 VA Palo Alto Healthcare System, Menlo Park, California, United States; 4 Veterans Health Administration, Palo Alto, California, United States

## Abstract

With the proliferation of virtual care, healthcare systems are exploring ways to bridge the digital divide among vulnerable patients. Department of Veterans Affairs (VA) is distributing devices for qualifying Veterans to enable video visits with medical providers at home, yet their use among older patients is unknown. This retrospective cohort study used administrative data to characterize the use of VA-loaned iPads among older Veterans compared to younger Veterans and identify demographic predictors of utilization. Among 16,385 patients who were shipped a VA-loaned iPad in 2014-2019, 33.66% (n=5,516) were over 65 years old, and 3.1% (n=503) were over the age of 85. Two thirds (n=6799) of younger patients had a video visit (mean=3 visits) with provider using iPad in the 6 months since shipment, compared to 50% (n=253) of 85+ year-olds (mean=1.8 visits). Most common types of virtual visits for the oldest old patients were for geriatrics or home-based primary care, compared to mental health visits among younger patients. Logistic regression identified characteristics of older patients who were more likely to use iPads, such a marital status, urban location, and lower disease burden, which is similar to their younger counterparts. While older age groups used VA-loaned tablets less frequently, those who engaged with the devices were similar in demographics as their younger counterparts. Older patients used iPads differently, with higher engagement in geriatric and primary care services. Providing devices for virtual care may allow health systems to more easily reach older patients in the comfort of their home.

